# Supplementary material for: Isolation, Identification and Genetic Characterization of Antibiotic Resistant Escherichia coli from Frozen Chicken Meat Obtained from Supermarkets at Dhaka City in Bangladesh
Source: Antibiotics (Basel). 2022 Dec 27;12(1):41. doi: 10.3390/antibiotics12010041 (PMC9855094; doi:10.3390/antibiotics12010041)
Supplement: Supplementary file 1 [file antibiotics-12-00041-s001.zip › antibiotics-2096799-supplementary.pdf]

Supplementary Table S1. Antimicrobial resistance patterns of *E. coli* (n=50).

No. of antibiotic	Antimicrobial resistance pattern	No. of antimicrobial classes in pattern (MDR)[a]	No. of isolate (%)
1 (3 isolates)	Sul	1(no)	1(2.00)
	Str	1(no)	1(2.00)
	Gen	1(no)	1(2.00)
2 (2 isolates)	Tet-Chl	2(no)	1(2.00)
	Ery-Chl	2(no)	1(2.00)
3 (19 isolates)	Tet-Ery-Sul	3(yes)	2(4.00)
	Gen-Amp-Ery	3(yes)	1(2.00)
	Str-Tet-Amp	3(yes)	1(2.00)
	Tet-Amp-Ery	3(yes)	2(4.00)
	Str-Ery-Sul	3(yes)	2(4.00)
	Str-Tet-Sul	3(yes)	2(4.00)
	Str-Gen-Amp	2(no)	1(2.00)
	Ery-Chlo-Sul	3(yes)	1(2.00)
	Str-Chlo-Sul	3(yes)	1(2.00)
	Gen-Tet-Ery	3(yes)	1(2.00)
	Gen-Amp-Ery	3(yes)	1(2.00)
	Str-Gen-Ery	2(no)	1(2.00)
	Gen-Ery-Sul	3(yes)	1(2.00)
	Gen-Tet-Amp	3(yes)	1(2.00)
	Str-Gen-Chlo	2(no)	1(2.00)
4 (17 isolates)	Str-Tet-Ery-Sul	4(yes)	2(4.00)
	Str-Gen-Tet-Ery	3(yes)	1(2.00)
	Tet-Amp-Ery-Sul	4(yes)	4(8.00)
	Str-Gen-Chlo-Sul	3(yes)	1(2.00)
	Gen-Amp-Ery-Chl	4(yes)	1(2.00)
	Gen-Tet-Chl-Sul	4(yes)	1(2.00)
	Str-Gen-Tet-Sul	3(yes)	2(4.00)
	Str-Tet-Amp-Chl	4(yes)	1(2.00)
	Str-Tet-Amp-Ery	4(yes)	1(2.00)
	Str-Tet-Chl-Sul	4(yes)	1(2.00)
	Str-Ery-Chl-Sul	4(yes)	1(2.00)
	Gen-Tet-Amp-Ery	4(yes)	1(2.00)
5 (6 isolates)	Str-Tet-Amp-Chl-Sul	5(yes)	1(2.00)
	Str-Tet-Amp-Ery-Sul	5(yes)	2(4.00)
	Str-Gen-Tet-Ery-Sul	4(yes)	1(2.00)
	Str-Gen-Tet-Amp-Ery	4(yes)	1(2.00)
	Str-Gen-Tet-Ery-Chl	4(yes)	1(2.00)
6 (2 isolates)	Str-Gen-Tet-Amp-Chl-Sul	5(yes)	1(2.00)
	Str-Gen-Tet-Amp-Ery-Sul	5(yes)	1(2.00)
7 (1 isolate)	Str-Gen-Tet-Amp-Ery-Chl-Sul	6(yes)	1(2.00)

[a] an isolate is defined as multidrug-resistant when it shows resistance to >2 classes of antimicrobial agents

Str: Streptomycin, Gen: Gentamicin, Tet: Tetracycline, Amp: Ampicillin, Erythromycin, Chl: Chloramphenicol, Sul: Sulfonamide

Supplementary Table S2. Pairwise association analysis of phenotypic antimicrobial resistance patterns.

Outcome (O)	O+	Predictor (P)	P+	O+/P+ (a)	O+/P- (b)	O-/P+ ©	O-/P- (d)	Odds Ratio	95% CI	p
Streptomycin	29	Gentamycin	22	13	16	9	12	1.08	0.35-3.36	0.88
		Tetracycline	33	20	9	13	8	1.37	0.42-4.45	0.6
		Ampicillin	22	11	18	11	10	0.55	0.18-1.73	0.31
		Erythromycin	31	15	14	16	5	0.33	0.10-1.16	0.08
		Chloramphenicol	15	10	19	5	16	1.68	0.48-5.95	0.42
		Sulfonamide	29	19	10	10	11	2.09	0.66-6.59	0.21
Gentamycin	22	Tetracycline	33	13	9	20	8	0.58	0.18-1.88	0.36
		Ampicillin	22	10	12	12	16	1.11	0.36-3.42	0.85
		Erythromycin	31	13	9	18	10	0.8	0.25-2.53	0.71
		Chloramphenicol	15	7	15	8	20	1.17	0.35-3.93	0.8
		Sulfonamide	29	9	13	20	8	0.28	0.09-0.90	0.03
Tetracycline	33	Ampicillin	22	18	15	4	13	3.9	1.05-14.51	0.04
		Erythromycin	31	21	12	10	7	1.2	0.37-4.06	0.71
		Chloramphenicol	15	8	25	7	10	0.46	0.13-1.60	0.22
		Sulfonamide	29	21	12	8	9	1.97	0.60-6.46	0.26
Ampicillin	22	Erythromycin	31	16	6	15	13	2.31	0.70-7.65	0.17
		Chloramphenicol	15	5	17	10	18	0.53	0.15-1.87	0.32
		Sulfonamide	29	10	12	19	9	0.39	0.12-1.25	0.12
Erythromycin	31	Chloramphenicol	15	6	25	9	10	0.27	0.08-0.95	0.04
		Sulfonamide	29	18	13	11	8	1.01	0.32-3.20	0.99
Chloramphenicol	15	Sulfonamide	29	9	6	20	15	1.13	0.33-3.85	0.85

Supplementary Table S3. Pairwise association analysis antimicrobial resistance genes (ARGs).

Outcome	O+	Predictor	P+	O+/P+ (a)	O+/P- (b)	O-/P+ ©	O-/P- (d)	Odds Ratio	95% CI	p
aadA1	28	*aac(3)-IV*	22	13	15	9	13	1.25	0.4049 to 3.8703	0.70
		tet(A)	33	19	9	14	8	1.21	0.3721 to 3.9113	0.76
		tet(B)	30	17	11	13	9	1.07	0.3425 to 3.3424	0.91
		bla_{CITM}	24	13	15	11	11	0.87	0.2833 to 2.6511	0.80
		bla_{SHV}	20	12	16	8	14	1.31	0.4170 to 4.1313	0.64
		ereA	32	15	13	17	5	0.34	0.0979 to 1.1767	0.09
		cmlA	17	9	19	8	14	0.83	0.2557 to 2.6877	0.75
		Cat1	18	13	15	5	17	2.95	0.8499 to 10.2167	0.09
		Sul1	28	17	11	11	11	1.55	0.4999 to 4.7774	0.44
aac(3)-IV	22	*tet(A)*	33	14	8	19	9	0.83	0.2557 to 2.6877	0.75
		tet(B)	30	13	9	17	11	0.94	0.2992 to 2.9198	0.91
		bla_{CITM}	24	13	9	11	17	2.23	0.7146 to 6.9737	0.17
		bla_{SHV}	20	10	12	10	18	1.5	0.4793 to 4.6948	0.49
		ereA	32	13	9	19	9	0.68	0.2140 to 2.1880	0.52
		cmlA	17	5	17	12	16	0.39	0.1127 to 1.3644	0.14
		Cat1	18	7	15	11	17	0.72	0.2228 to 2.3349	0.59
		Sul1	28	10	12	18	10	0.46	0.1479 to 1.4490	0.19
tet(A)	33	*tet(B)*	30	20	13	10	7	1.08	0.3270 to 3.5465	0.90
		bla_{CITM}	24	16	17	8	9	1.06	0.3280 to 3.4177	0.92
		bla_{SHV}	20	14	19	6	11	1.35	0.4025 to 4.5339	0.62
		ereA	32	21	12	11	6	0.94	0.2813 to 3.2388	0.94
		cmlA	17	11	22	6	11	0.92	0.2679 to 3.1367	0.89
		Cat1	18	11	22	7	10	0.71	0.2136 to 2.3881	0.58
		Sul1	28	20	13	8	9	1.73	0.5313 to 5.6379	0.36
tet(B)	30	*bla_{CITM}*	24	15	15	9	11	1.22	0.3928 to 3.8027	0.73
		bla_{SHV}	20	13	17	7	13	1.42	0.4414 to 4.5697	0.56
		ereA	32	19	11	13	7	0.93	0.2854 to 3.0311	0.90

		cmlA	17	12	18	5	15	2	0.5740 to 6.9681	0.28
		Cat1	18	11	19	7	13	1.08	0.3299 to 3.5040	0.90
		Sul1	28	19	11	9	11	2.11	0.6670 to 6.6818	0.20
bla_{CITM}	24	bla_{SHV}	20	10	14	10	16	1.14	0.3682 to 3.5472	0.82
		ereA	32	17	7	15	11	1.78	0.5501 to 5.7657	0.34
		cmlA	17	7	17	10	16	0.66	0.2019 to 2.1501	0.49
		Cat1	18	9	15	9	17	1.13	0.3568 to 3.5999	0.83
		Sul1	28	8	16	20	6	0.15	0.0431 to 0.5215	0.002
bla_{SHV}	20	*ereA*	32	13	7	19	11	1.08	0.3299 to 3.5040	0.90
		cmlA	17	6	14	11	19	0.74	0.2206 to 2.4845	0.63
		Cat1	18	10	10	8	22	2.75	0.8341 to 9.0662	0.10
		Sul1	28	9	11	19	11	0.47	0.1497 to 1.4992	0.20
ereA	32	*cmlA*	17	9	23	8	10	0.49	0.1462 to 1.6363	0.25
		Cat1	18	9	23	9	9	0.39	0.1175 to 1.3033	0.13
		Sul1	28	18	14	10	8	1.03	0.3215 to 3.2904	0.96
cmlA	17	*Cat1*	18	9	8	9	24	3	0.8837 to 10.1847	0.08
		Sul1	28	10	7	18	15	1.19	0.3643 to 3.8908	0.77
Cat1	18	*Sul1*	28	12	6	16	16	2	0.6022 to 6.6419	0.26
